# Determinants of vaccination coverage during the COVID-19 pandemic among children aged 12–23 months in southern Ethiopia: a cross-sectional study

**DOI:** 10.3389/fped.2025.1566443

**Published:** 2025-08-22

**Authors:** Tigist Enyew Gedamu, Anmut Assemie, Leyla Temam Aleye, Meskerem Teketel Woldemariam, Tsegaye Gebre Argago, Alula Seyum Buda

**Affiliations:** ^1^ Department of Pediatrics and Child Health Nursing, Wachemo University, Hosanna, Ethiopia; ^2^ Department of Biology, Wachemo University, Hosanna, Ethiopia; ^3^ Department of Biomedical Science, Hawassa University, Hawassa, Ethiopia; ^4^ Department of Comprehensive Nursing, Wachemo University, Hosanna, Ethiopia; ^5^ Department of Surgical Nursing, Wachemo University, Hosanna, Ethiopia

**Keywords:** COVID-19, pandemic, full immunization, vaccination coverage, children, Ethiopia, cross-sectional study

## Abstract

**Background:**

Millions of children, particularly in low and middle-income countries, are deprived of a comprehensive vaccination schedule. The advent of the COVID-19 pandemic exacerbated this issue by significantly disrupting vaccination schedules and other critical health initiatives. In light of this challenge, our study sought to evaluate vaccination coverage and identify its determinants among children aged 12–23 months in southern Ethiopia during the COVID-19 pandemic.

**Methods:**

We conducted a community-based cross-sectional study. Three kebeles were purposively selected, with 415 households with 12–23-month-old children from each kebele selected using a systematic sampling technique. Data were collected using an adapted Ethiopian 2019 Mini Demographic Health Survey immunization coverage tool. Data were entered, cleaned, and checked using EpiData version 3.1 and analyzed using SPSS version 24. Bivariate and multivariable logistic regression analyses were performed. Variables with a *P*-value of <0.05 were considered significant determinants of full vaccination coverage.

**Results:**

Our study found that the full vaccination coverage rate among children in the study area was 44.5% (95% CI = 39.8–49.4). Households with two children were nearly twofold more likely to be fully vaccinated (AOR = 1.99, 95% CI = 1.01–3.95, *P*-value = 0.047), children whose caregivers reported traveling to the nearest immunization site were nearly threefold more likely to be fully vaccinated (AOR = 2.78, 95% CI = 1.38–5.57, *P*-value = 0.004,), children who underwent multiple immunization schedules were less likely to be fully vaccinated (AOR = 0.068, 95% CI = 0.035–0.134, *P*-value = 0.000), and children whose caregivers reported receiving information from television were twice as likely to be fully vaccinated (AOR = 2.02, 95% CI: 1.05–3.89).

**Conclusions:**

In summary, our findings indicate a pressing need to address the identified determinants to improve vaccination coverage, which is currently below the World Health Organization's recommended levels. Therefore, targeted efforts must be made to enhance awareness and accessibility, particularly focusing on family size, logistical barriers, multiple immunization schedules, and effective communication through various media channels. Meeting the recommended vaccination rate will require coordinated action among healthcare providers, policymakers, and communities.

## Introduction

Most countries in the world experienced immunization disruption during the initial phase of the COVID-19 pandemic (1). Global vaccination coverage declined from 90% to 86% between 2019 and 2021 compared to between 2015 and 2019 (2). Childhood vaccination coverage declined from 95% to nearly 90% compared to previous years in the United States before the COVID-19 pandemic (3). A substantial immunization disruption was also seen in countries where COVID-19 emerged (4). The decrease in the vaccination coverage ranged from 1.3% to 7.8% and the percentage of children with no vaccinations remained low at 1.2% (5). Millions of children in low and middle-income countries did not receive their full series of vaccines (6). Vaccinations and other child health services in low and middle-income countries were disrupted following the COVID-19 pandemic (7). Globally, measles outbreaks increased following the emergence of the COVID-19 pandemic (1), leading to infant death from its complications due to 70%–80% routine vaccine disruption (5). The rate of unvaccinated children increased from 0.8% to 1.1% between 2016 and 2017 (8). More than 117 million children did not receive a measles vaccine during the COVID-19 pandemic, and 2.3 million died each year from measles outbreaks (9).

Worldwide, only 180 children have tested positive for COVID-19, while approximately 62,000 children die annually due to vaccine-preventable diseases in Ethiopia (7). Nearly 60% of children were fully vaccinated in low and middle-income countries, with a zero-dose prevalence ranging from 12.5% to 3.4% in the poorest and wealthiest countries, respectively (10). Discrepancies in full vaccination coverage have been reported in Ghana, Nigeria, Kenya, and Ethiopia, with 89.5%, 78.9%, 66.4%, and 52.4% and 38% full vaccination coverage, respectively (4, 11–14). Previous studies in Ethiopia showed a full vaccination coverage of 58.4% and 42.2%, which is lower than the WHO-recommended vaccination coverage (4, 6). Zero-dose prevalence was reported to be 1.0%, 16.5%, 19.1%, 40%, 8.4%, 14%, and 24.6% in previous studies of China (11), sub-Saharan Africa (15), Congo (16), Nigeria (17), and Ethiopia, respectively (4, 6, 12). In general, disparities in vaccination coverage have been found in different research studies to be a result of the COVID-19 pandemic and other determinants before the pandemic (3, 18–20). Therefore, catch-up vaccination schedules should be designed after identifying children who have missed routine vaccination following a pandemic such as the COVID-19 pandemic(1, 3). Thus, our study will provide input for health professionals to strengthen the existing expanded program of immunization (EPI) and contribute towards eliminating and minimizing missed opportunities and interruptions in vaccinations following any future pandemic. In addition, this study will provide supplementary data to zonal, regional, and national policymakers so that they take immediate action on catch-up vaccinations despite the occurrence of any future pandemic. Therefore, this study was conducted to determine the routine vaccination coverage and its determinant factors among the study participants during the COVID-19 pandemic.

## Methods and materials

### Study area, period, and design

In the period from August to September 2021, we conducted a community-based cross-sectional study in Hosanna Town, located in the former Southern Nations, Nationalities, and Peoples' Region (SNNPR) of Ethiopia, currently called the Central Ethiopia Region. The town is situated approximately 230 km from Addis Ababa, the capital of Ethiopia. Hosanna has a total population of 82,711 residents, comprising 41,153 female residents and 41,558 male residents. Among this population, there are approximately 11,052 children under the age of 5 years; this group includes 5,559 girls and 5,493 boys (21). Furthermore, vaccination coverage within the SNNPR region stands at only 38%, and approximately 24.2% of children remain unvaccinated (22).

### Source population

All mothers with a baby aged 12–23 months during the study period in Hossana Town.

### Study population

All mothers with a baby aged 12–23 months in the selected kebeles who fulfilled the inclusion criteria.

### Inclusion and exclusion criteria

All mothers with a 12–23-month-old child who had lived at least for 1 year in the selected study areas were included, while those with a serious illness during the study period were excluded.

### Sample size determination and sampling procedure

The sample size for the study was determined using the single population proportion formula, which took into account a vaccination coverage rate of 43% ascertained from prior research (22). This approach provided the largest necessary sample size, calculated at a 95% confidence level with the margin of error set at 0.05. Ultimately, the final sample size was adjusted to 415 participants after factoring in an anticipated non-response rate of 10%.

To ensure equitable representation within the research framework, three specific kebeles with large populations of prospective study participants were purposefully selected from a total of six kebeles in Hossana Town, as this increased the generalizability and validity of our study. As such, the sample sizes were allocated as follows: 120 for Arada, 133 for Lichamba, and 162 for Heto Kebele. The initial household was chosen randomly, with subsequent households selected using a systematic sampling at every ninth unit (*k* = 9), which was determined based on an initial source population of 3,744 and the established sample size of 415.

### Study variables

This study considered vaccination coverage to be the dependent variable and maternal sociodemographic variables (age, sex, education level, income, religion, residence, etc.), maternal healthcare service utilization, child information (age, sex, birth weight), child vaccination history (vaccinated, not vaccinated, fully vaccinated, partially vaccinated, etc.) and COVID-19 pandemic-related variables (full lockdown of immunization services, physical distancing measures, stay-at-home messaging, mothers’ and caregivers’ fear of visiting health facilities, closing of borders limiting vaccination service access, diversion of resources for the pandemic, postponed vaccination campaigns, etc.) to be the independent variables.

### Data collection instrument and procedure

The Ethiopian Mini Demographic Health Survey 2019 (EmDHS-2019) tool (22) was meticulously adapted to create a comprehensive questionnaire that addressed various dimensions of maternal and child health. Specifically, it included inquiries regarding maternal sociodemographic characteristics, utilization of maternal healthcare services, detailed child information, vaccination history, and variables pertinent to COVID-19 (see Supplementary Material).

To enhance the accuracy of the vaccination coverage assessment, the data collectors utilized the children's immunization cards as the primary source of verification. This methodological approach ensures that the data gathered reflect actual immunization statuses rather than relying solely on parental recall or reporting. The implementation of this survey required the recruitment of six nurses with bachelor's degrees as data collectors and three nurses with master's degrees as supervisors. Such a structured team composition facilitated robust data collection procedures while ensuring adherence to high standards throughout the process. The overall supervision was conducted by the principal investigator (TE), whose oversight was integral to maintaining methodological consistency and ensuring that the objectives were met effectively.

### Measurements

Vaccination coverage was categorized as fully vaccinated or partially vaccinated. Full vaccination coverage was categorized as having received all the EPI-recommended vaccines at birth, 6 weeks, 10 weeks, 14 weeks, and 9 months. A child who received the BCG, OPV0, OPV1, OPV2, OPV3, DPT1, DPT2, DPT3, PCV1, PCV2, PCV3, RORA1, ROTA2, and measles vaccines on the appropriate immunization schedule was deemed to be fully immunized. Partial vaccination coverage was categorized as having received one of the EPI-recommended vaccines on the appropriate immunization schedule. Vaccination status was cross-checked with the child's vaccination card.

### Data processing and analysis

Data were entered, cleaned, and checked using EpiData version 3.1 and analyzed using SPSS version 24.

Descriptive statistics, such as frequencies and percentages, were computed to describe the research variables. Bivariate and multivariable logistic regression analyses were performed to identify predictors. The variance inflation factor and the Hosmer–Lemeshow goodness of fit test were used to check the model’s stability and its goodness of fit. After controlling for other confounders and adjusting for likely confounders, variables with a *p*-value of <25% in the bivariate logistic analysis were included in the multivariate logistic analysis. Variables with a *p*-value of <0.05 at a 95% confidence level were considered significant determinants of the outcome variable. Finally, the results are presented in the text and tables.

## Results

### Sociodemographic characteristics

In total, 220 (52.9%), 214 (51.4%), and 271 (66.3%) children were in the age group of 12–18 months, were male, and had attended four or more ANC visits, respectively. The date of birth of the majority of children was verified from their immunization card (397, 95.4%) and the majority needed to travel more than 30 min to receive their vaccinations (398, 95.6%) (Table 1).

**Table 1:** Socio-demographic characteristics of children aged 12–23 months in southern Ethiopia, 2021.

Variable	Category	*N* (%)
Sex	Male	214 (51.4)
	Female	202 (48.6)
Age	19–23 months	184 (44.2)
	12–18 months	220 (52.9)
Birth order	First	163 (39.2)
	Second	109 (26.2)
	Third	93 (22.4)
	Fourth and older	51 (12.3)
Primary care taker able to read or write	Yes	355 (85.3)
	No	61 (14.7)
Primary care taker attend formal school	Yes	353 (84.9)
	No	63 (15.18)
Birth date of child verified with card	Yes	397 (95.4)
	No	19 (4.6)
Number of ANC visit	First	2 (0.5)
	Second	23 (5.6)
	Third	113 (27.6)
	Fourth and more	271 (66.3)
Age of primary care taker	>36 years	128 (30.8)
	<35 years	288 (69.2)
Distance from the nearest health post	>30 min	400 (96.2)
	<30 min	16 (3.8)
Distance from the nearest health center	>30 min	398 (95.7)
	<30 min	18 (4.3)
Distance from the nearest immunization site	>30 min	398 (95.6)
	<30 min	18 (4.3)

### Vaccination coverage

In total, 185 (44.5%) children were fully vaccinated, while most children (230, 55.3%) were partially immunized, even though the majority (338, 81.3%) had a vaccination card. The main reasons for not being fully immunized were lack of vaccines (104, 25.0%) during the immunization session, physical distancing measures (114, 27.4%), stay-at-home messaging (116, 27.9%), and the mother’s fear of visiting the health facility (117, 28.1%) during the COVID19 pandemic (Tables 2 and 3).

**Table 2:** Vaccination coverage for routine vaccines among children 12–23months in southern Ethiopia, 2021.

Variable	Yes *N* (%)	No *N* (%)
Child have vaccination card	338 (81.3)	78 (18.8)
BCG vaccination	332 (79.8)	84 (20.2)
OPV0	334 (80.3)	82 (19.7)
OPV1	334 (80.3)	82 (19.7)
OPV2	334 (80.3)	82 (19.7)
OPV3	335 (80.5)	81 (19.5)
PENTA1	296 (71.2)	120 (28.8)
PENTA2	297 (71.4)	119 (28.6)
PENTA3	298 (71.6)	118 (28.4)
PCV1	287 (69.0)	129 (31.0)
PCV2	291 (70.0)	125 (30.0)
PCV3	293 (70.4)	123 (29.6)
ROTA1	287 (69.0)	129 (31.0)
ROTA2	283 (68.0)	133 (32.0)
Measles	323 (77.6)	93 (22.4)

BCG, Bacillus Calmette-Guerin; OPV, oral polio vaccine; PENTA, a prefix meaning “five,” Pneumococcal conjugate vaccines; ROTA, rotavirus vaccine.

**Table 3:** Vaccination status and factors affecting it among among children aged 12–23 months in southern Ethiopia, 2021.

Variable	Yes *N* (%)	No *N* (%)
Fully immunized	185 (44.5)	230 (55.3)
Immunization prevent childhood illness	413 (99.3)	3 (0.7)
Immunization starting <1 month	12 (2.9)	404 (97.1)
Number of immunization schedules <4	42 (10.1)	374 (89.9)
Child did not fully immunized due to the absence of vaccine	104 (25.0)	312 (75.0)
Child did not fully immunized due to mother busy	8 (1.9)	408 (98.1)
Child did not fully immunized due to physical distancing measures	114 (27.4)	302 (72.6)
Child did not fully immunized due to stay at home message	116 (27.9)	300 (72.1)
Child did not fully immunized due to fear of mother to visit health facility	117 (28.1)	299 (71.9)

### Factors predicting vaccination coverage

In the bivariate analysis, variables including receiving multiple immunization schedules; being the primary caregiver; learning about vaccine side effects from the television, newspaper, health worker, community event, or health extension worker; age of the child; having a vaccination card; husband living with the mother; birth order; place of routine vaccination; total live births; distance from the nearest immunization site; primary caregiver reading or writing ability; and date of birth verified using vaccination card were eligible for the multiple logistic regression with a *P*-value = <0.25 so as not to leave out epidemiologically important variables in the context of our study.

Among these variables, children from households with two children were more likely to be fully vaccinated than those from households with four or more children (AOR = 1.99, CI = 1.01–3.95, *P*-value = 0.047). Children who needed to travel less than 30 min to the nearest immunization site were more likely to have full vaccination coverage (AOR = 2.78, CI = 1.38–5.57, *P* = 0.004) than those who needed to travel more than 30 min. Receiving multiple immunization schedules was significantly negatively associated with full vaccination coverage (AOR= 0.068, CI = 0.04–0.13, *P* = 0.000) compared to receiving fewer. Having the television as a source of information about vaccine side effects increased the likelihood of full vaccination coverage by more than twofold (AOR = 2.022, 95% CI = 1.05–3.89, *P* = 0.035) compared to not receiving this information from the television (Table 4).

**Table 4:** Bivariate and multivariable analysis of factors predicting vaccination coverage among children aged 12–23 months in southern Ethiopia, 2021.

Variable		Vaccination status		*P*-value	COR (95% CI)	AOR (95% CI)
		Fully immunized *n* (%)	Partially immunized, *n* (%)			
Age of the child	12–18 months	112 (61.5)	108 (48.9)	0.061	1.67 (1.12–2.49)	1.79 (0.97–3.28)
	19–23 months	70 (38.5)	113 (51.1)		1	1
Number of total live births	Two	75 (50.3)	87 (48.6)	0.047	1.18 (0.73–1.92)	1.99 (1.01–3.95)*
	Three	55 (36.9)	54 (30.2)	0.296	0.58 (0.31–1.09)	0.62 (0.26–1.51)
	≥Four	19 (12.8)	38 (21.2)		1	1
Distance from the nearest immunization site	<30 min	142 (76.8)	128 (55.7)	0.004	2.63 (1.71–4.04)	2.78 (1.38–5.57)*
	>30 min	43 (23.2)	102 (44.3)		1	1
Number of immunization schedules	≥Four times	59 (31.9)	195 (84.8)	0.000	0.08 (0.05-0.14)	0.07 (0.04-0.13)*
	≤Four times	126 (68.1)	35 (15.2)		1	1
Husband living with	Yes	180 (97.83)	217 (94.76)	0.101	2.49 (0.79–7.85)	3.61 (0.78–16.74)
	No	4 (2.17)	12 (5.24)		1	1
Primary care taker able to read or write	Yes	23 (12.43)	37 (16.09)	0.088	1.35 (0.77–2.37)	0.46 (0.19–1.12)
	No	162 (87.57)	193 (83.91)		1	1
Place for routine vaccination	Health center	68 (36.76)	79 (34.96)	0.450	0.05 (0.01–0.39)	0.77 (0.39–1.51)
	Health post	1 (0.54)	21 (9.29)		1	1
Told vaccine side effect from television	Yes	147 (79.46)	129 (56.09)	0.035	3.03 (1.95–4.71)	2.02 (1.05–3.89)*
	No	38 (20.54)	101 (43.91)		1	1

AOR, adjusted odds ratio; COR, crude odds ratio; * significant at *P*-value ≤ 0.05.

## Discussion

This study aimed to assess the vaccination coverage among children aged 12–23 months and identify its predictors in a town in southern Ethiopia. The study revealed a full vaccination coverage rate of 45%. Several key factors were identified to determine immunization rates, including the total number of live births, the distance from the nearest immunization site, the number of immunization schedules received, and the source of information regarding vaccine side effects.

Notably, this study's vaccination coverage rate of 45% falls significantly short of the World Health Organization's recommended target for full vaccination coverage of more than 90%. In addition, the rate is lower than previous studies conducted in China (11), Nigeria (14), Sierra Leone (23), Ethiopia (10, 13, 24), and elsewhere in sub-Saharan Africa (25, 26). However, it surpasses the rates from specific regions such as Nigeria (34.4%) (27) and parts of Ethiopia (38%), including Afar (25.1%), and Bench Maji (42.2%) (4, 12, 15). This underscores the need for targeted interventions aimed at improving vaccination rates among young children. It is imperative that we work towards achieving higher immunization coverage in accordance with global health standards (7) and previous studies in the globe (2, 9, 19, 20, 28–32) by addressing barriers such as accessibility and enhancing public awareness regarding vaccines. Our results align with previous studies (33, 34), suggesting similar trends in vaccination coverage. This similarity in vaccination coverage may be due to measures taken to prevent the transmission of COVID-19 during the COVID-19 pandemic in developed countries (5, 35). Notably, measles vaccination coverage was higher (77.6%) in our results compared to other international and national reports (17, 35, 36), a difference potentially linked to vigorous preventive strategies against the transmission of COVID-19.

In our study, physical distancing measures, stay-at-home messaging, and the mother’s fear of visiting the health facility were COVID-19 pandemic-related factors related to partial vaccination coverage, as in previous studies (35, 37). However, none of them were significantly associated with full vaccination coverage. This may be related to protective measures, such as social distancing, mandatory mask-wearing, and sanitation procedures. In contrast, these variables predicted decreased immunization coverage in developed countries (4, 9, 37). These differences may be due to strict measures taken to control the COVID-19 pandemic in developed countries. In conclusion, the COVID-19 pandemic significantly decreased full vaccination coverage, which is supported by previous studies (1, 38, 39).

Being from a household with fewer children predicted full vaccination coverage, which is in line with the findings of previous studies (24, 40). Larger families may face logistical challenges in managing multiple schedules and financial constraints, which can hinder timely vaccinations and potentially lead to missed immunizations. Conversely, smaller families may find it easier to coordinate healthcare appointments, thus promoting higher vaccination rates. Therefore, understanding the dynamics of family size can help health authorities to design targeted strategies. Traveling a short distance to the nearest immunization site also predicted full vaccination coverage in this study, which was consistent with findings in studies conducted in other regions of the world (10, 14, 24, 41–45). Families residing in underserved areas may encounter significant barriers when seeking vaccinations for their children, such as limited transportation options or significant costs associated with travel. This is supported by the findings of a research study in Ethiopia (46). Therefore, public health initiatives that address the geographical barriers, such as mobile clinics or expanding clinic locations, can significantly enhance full vaccination accessibility.

In this study, receiving multiple immunization schedules significantly reduced full immunization coverage, a finding supported by a previous study in Sierra Leone that found decreased vaccination coverage when doses were administered sequentially (23). While we expected multiple immunization schedules to have a direct relationship with full immunization coverage, our results indicate a paradox. A study in Ethiopia also highlighted that not knowing whether to return for future visits can be attributed to not having full vaccination coverage (47). Receiving a vaccine at a specific time and undergoing multiple vaccination schedules do not have an equal impact on the full immunization coverage. Thus, clear, consistent, and detailed information about the necessity and timing of vaccinations can help caregivers to understand and adhere to vaccination schedules. Additionally, health professionals must communicate the risks of under-vaccination effectively to foster a more comprehensive understanding among parents. Moreover, a deeper examination to uncover the underlying factors contributing to the paradoxical trend of vaccine administration and immunization coverage is pertinent. Obtaining information about vaccine side effects from the television was found to be another predictor of full vaccination coverage, similar to previous reports (6, 16, 22, 27, 30, 40, 48). Access to reliable and accurate information is essential for parents to make informed decisions regarding their child's health. Those with limited access to alternative, accurate sources of health information may rely heavily on television broadcasts. Hence, television networks have a responsibility to provide evidence-based content.

The findings of our study imply that it is imperative to prioritize and address each of the above factors that influence vaccination uptake. These include accommodating decreased family sizes, ensuring proximity to immunization sites, ensuring strict adherence to multiple immunization schedules, and improving access to reliable information regarding vaccine side effects. By addressing these variables through targeted public health strategies, stakeholders can foster an environment that promotes vaccination adherence, ultimately contributing to the overall health and wellbeing of communities. It is important to acknowledge that our study was conducted within a specific community, and the purposeful selection of the kebeles may limit the generalizability of some of our findings and may lead to selection bias. In addition, causality between the predictor variables and the outcome variable could not be determined, as we employed a cross-sectional design, which may limit the broader applicability of the results. Furthermore, parental reporting may have led to recall bias. Therefore, future research should consider various demographic and geographical contexts to enhance generalizability and inform effective public health strategies aimed at improving vaccination rates.

## Conclusions and recommendations

Our study shows that the full vaccination coverage rate during the COVID-19 pandemic in the study area was only 45%, significantly lower than the WHO’s global target. Several factors contributed to this decreased immunization rate, including the implementation of physical distancing measures, caregivers' apprehension to take their children to vaccination sites, and stay-at-home directives. Furthermore, concerns regarding vaccine side effects, smaller family sizes, and distance from the nearest immunization site emerged as predictive factors affecting vaccination uptake. To effectively address these challenges and bridge the existing gaps in vaccination coverage, it is essential to focus on the identified determinants. Many lessons have been learned from the COVID-19 pandemic that should inform plans to minimize the impact on vaccination coverage in future pandemics. It is essential to design effective outreach and communication strategies in order to enhance vaccination coverage during future pandemics. Therefore, comprehensive community engagement initiatives, such as collaborating with local health authorities, schools, and community organizations, can facilitate trust building between healthcare providers and children's legal guardians, which is supported by previous studies (1, 49, 50). In addition, mobile vaccination clinics and school-based immunization programs are crucial to reduce barriers related to distance from the nearest immunization site. In conclusion, strategies are required to achieve the World Health Organization's recommended levels of vaccination coverage. Such initiatives must prioritize accessible information and simultaneously address concerns related to vaccine safety and accessibility.

## Data Availability

The raw data supporting the conclusions of this article will be made available by the authors, without undue reservation.

## References

[1] Ota MOB. Impact of COVID-19 pandemic on routine immunization. Ann Med. (2021) 53(1):2286–97. 10.1080/07853890.2021.2009128 34854789 PMC8648038

[2] Wang Q Leung K Jit M Wu JT Lin L. Global socioeconomic inequalities in vaccination coverage, supply, and confidence. NPJ Vaccines. (2025) 10(1):91. 10.1038/s41541-025-01143-8 40346086 PMC12064651

[3] Seither R Yusuf OB Dramann D Calhoun K Mugerwa-Kasujja A Knighton CL Coverage with selected vaccines and exemption rates among children in kindergarten—United States, 2023–24 school year. MMWR Morbidity and Mortality Weekly Report. (2024) 73(41):925–32. 10.15585/mmwr.mm7341a3 39418212 PMC11486350

[4] Meleko A Geremew M Birhanu F. Assessment of child immunization coverage and associated factors with full vaccination among children aged 12–23 months at Mizan Aman Town, Bench Maji Zone, Southwest Ethiopia. Int J Pediatr. (2017) 2017:7976587. 10.1155/2017/7976587 29434643 PMC5757163

[5] Hill HA Yankey D Elam-Evans LD Mu Y Chen M Peacock G Decline in vaccination coverage by age 24 months and vaccination inequities among children born in 2020 and 2021—national immunization survey-child, United States, 2021–2023. MMWR Morb Mortal Wkly Rep. (2024) 73(38):844–53. 10.15585/mmwr.mm7338a3 39325676 PMC11563569

[6] Tesfaye TD Animaw TW Kasa AS. Vaccination coverage and associated factors among children aged 12–23 months in northwest Ethiopia. Hum Vaccin Immunother. (2018) 14(10):2348–54. 10.1080/21645515.2018.1502528 30118398 PMC6284506

[7] Getnet M Jejaw M Belachew TB Addis B Dellie E Tafere TZ Incomplete basic vaccination and associated factors among children aged 12–23 months in resource-limited countries: a spatial and multilevel regression analysis of recent DHS data from 48 countries. Front Public Health. (2025) 13:1463303. 10.3389/fpubh.2025.1463303 40297029 PMC12036240

[8] Anderson KM Creanza N. Internal and external factors affecting vaccination coverage: modeling the interactions between vaccine hesitancy, accessibility, and mandates. (2023) 3(10):e0001186. 37792691 10.1371/journal.pgph.0001186 PMC10550134

[9] UNICEF. Balancing the needs for child health during COVID-19. UNICEF for every child, Ethiopia. PLOS Global Public Health. (2020).

[10] Cata-Preta BO Santos TM Mengistu T Hogan DR Barros AJD Victora CG. Zero-dose children and the immunisation cascade: understanding immunisation pathways in low and middle-income countries. Vaccine. (2021) 39(32):4564–70. 10.1016/j.vaccine.2021.02.072 33744046 PMC8314014

[11] Adokiya MN Baguune B. Evaluation of immunization coverage and its associated factors among children 12–23 months of age in Techiman Municipality, Ghana, 2016. (2017) 75:28. 28652913 10.1186/s13690-017-0196-6 PMC5483840

[12] Bogale B Tiruneh GT Belete N Hunegnaw BM Fesseha N Zergaw TS Risk factors associated with zero-dose and under-immunized children, and the number of vaccination doses received by children in Ethiopia: a negative binomial regression analysis. BMC Public Health. (2025) 25(1):1693. 10.1186/s12889-025-22837-7 40335968 PMC12057091

[13] Hailu S Astatkie A Johansson KA Lindtjørn B. Low immunization coverage in Wonago district, southern Ethiopia: a community-based cross-sectional study. PLoS One. (2019) 14(7):e0220144. 10.1371/journal.pone.0220144 31339939 PMC6655723

[14] Eze P Agu UJ Aniebo CL Agu SA Lawani LO Acharya Y. Factors associated with incomplete immunisation in children aged 12–23 months at subnational level, Nigeria: a cross-sectional study. BMJ Open. (2021) 11(6):e047445. 10.1136/bmjopen-2020-047445 34172548 PMC8237740

[15] Ozigbu CE Olatosi B. Correlates of zero-dose vaccination Status among children aged 12–59 months in sub-Saharan Africa: a multilevel analysis of individual and contextual factors. Vaccines. (2022) 10(7):1052. 35891216 10.3390/vaccines10071052 PMC9322920

[16] Ishoso DK Danovaro-Holliday MC. “Zero dose” children in the democratic republic of the Congo: how many and who are they? Vaccines. (2023) 11(5):900. 37243004 10.3390/vaccines11050900 PMC10224070

[17] Sato R. Zero-dose, under-immunized, and dropout children in Nigeria: the trend and its contributing factors over time. Vaccines (Basel). (2023) 11(1):181. 10.3390/vaccines11010181 36680025 PMC9862777

[18] Koskei A. Factors associated with discrepancies in vaccination and immunization coverage of measles, tetani and hepatitis B among children aged 12–23 months in Narok County, Kenya. medRxiv [Preprint]. 2025-03 (2025). Available online at: 10.1101/2025.03.04.25323382

[19] Zhou Y Li D Cao Y Lai F Wang Y Long Q Immunization coverage, knowledge, satisfaction, and associated factors of non-national immunization program vaccines among migrant and left-behind families in China: evidence from Zhejiang and Henan provinces. Infect Dis Poverty. (2023) 12(1):93. 37833775 10.1186/s40249-023-01145-5 PMC10571434

[20] Phillips DE Dieleman JL Lim SS Shearer J. Determinants of effective vaccine coverage in low and middle-income countries: a systematic review and interpretive synthesis. BMC Health Serv Res. (2017) 17(1):681. 10.1186/s12913-017-2626-0 28950899 PMC5615444

[21] QOTERA.ORG. Ethiopian Census—2017 Projection – SNNP – Hadiya – Hosaena. (2019). Available online at: https://www.qotera.org/en-US/2017/snnp/hadiya/hosaena/.2019 (Accessed 27, 2020).

[22] Ethiopian Public Health Institute. Ethiopia mini Demographic and Health Survey 2019: Key Indicators. Rockville, Maryland, USA: EPHI and 297 ICF (2019).

[23] Wassenaar M Fombah AE Chen H Owusu-Kyei K Williams J Sunders JC Immunisation coverage and factors associated with incomplete immunisation in children under two during the COVID-19 pandemic in Sierra Leone. BMC Public Health. (2024) 24(1):143. 10.1186/s12889-023-17534-2 38200476 PMC10777622

[24] Cutts FT Danovaro-Holliday MC Rhoda DA. Challenges in measuring supplemental immunization activity coverage among measles zero-dose children. Vaccine. (2021) 39(9):1359–63. 10.1016/j.vaccine.2020.11.050 33551302 PMC7903240

[25] Costa JC Weber AM Darmstadt GL Abdalla S Victora CG. Religious affiliation and immunization coverage in 15 countries in Sub-Saharan Africa. Vaccine. (2020) 38(5):1160–9. 10.1016/j.vaccine.2019.11.024 31791811 PMC6995994

[26] Bangura JB Xiao S Qiu D Ouyang F Chen L. Barriers to childhood immunization in sub-Saharan Africa: a systematic review. BMC Public Health. (2020) 20:1–15. 10.1186/s12889-020-09169-4 32664849 PMC7362649

[27] Nguyen KH Zhao R. Trends in vaccination schedules and up-to-date status of children 19–35 months, United States, 2015–2020. Vaccine. (2023) 41(2):467–75. 10.1016/j.vaccine.2022.11.023 36481107

[28] Gualu T Dilie A. Vaccination coverage and associated factors among children aged 12–23 months in Debre Markos town, Amhara regional state, Ethiopia. Adv Public Health. (2017) 2017(1):5352847. 10.1155/2017/5352847

[29] Kassahun MB Biks GA Teferra AS. Level of immunization coverage and associated factors among children aged 12–23 months in Lay Armachiho District, North Gondar Zone, Northwest Ethiopia: a community based cross sectional study. BMC Res Notes. (2015) 8:1–10. 10.1186/s13104-015-1192-y 26071403 PMC4467673

[30] Noh JW Kim YM Akram N Yoo KB Park J Cheon J Factors affecting complete and timely childhood immunization coverage in Sindh, Pakistan: a secondary analysis of cross-sectional survey data. PLoS One. (2018) 13(10):e0206766. 10.1371/journal.pone.0206766 30379947 PMC6209382

[31] Ketema DB Assemie MA Alamneh AA Alene M Chane KY Alamneh YM Full vaccination coverage among children aged 12–23 months in Ethiopia: a systematic review and meta-analysis. BMC Public Health. (2020) 1(10):777. 10.1186/s12889-020-08940-x PMC7249262 32448220

[32] Eshete A Shewasinad S Hailemeskel S. Immunization coverage and its determinant factors among children aged 12–23 months in Ethiopia: a systematic review, and meta-analysis of crosssectional studies. BMC Pediatrics. (2020) 20(1):283. 10.1186/s12887-020-02163-0 32513135 PMC7278125

[33] Bramer CA. Decline in child vaccination coverage during the COVID-19 pandemic—Michigan care improvement registry, May 2016–May 2020. MMWR Morb Mortal Wkly Rep. (2020) 69(20):630–1. 10.15585/mmwr.mm6920e1 32437340

[34] Nour TY Farah AM Ali OM Abate KH. Immunization coverage in Ethiopia among 12–23 month children: systematic review and meta-analysis. MC Public Health. (2020) 20(1):1134. 10.1186/s12889-020-09118-1 PMC7370412 32689962

[35] McDonald HI. Early impact of the coronavirus disease (COVID-19) pandemic and physical distancing measures on routine childhood vaccinations in England, January to April 2020. Eurosurveillance. (2020) 25(19):2000848. 10.2807/1560-7917.ES.2020.25.19.2000848 32431288 PMC7238742

[36] Alemayehu A Tefera S. Measles second dose vaccination coverage and associated factors in Ethiopia: a systematic review and meta-analysis. Vaccine: X. (2025) 7:100675. 10.1016/j.jvacx.2025.100675

[37] Nelson R. COVID-19 disrupts vaccine delivery. Lancet Infect Dis. (2020) 20(5):546. 10.1016/S1473-3099(20)30304-2 32311326 PMC7164887

[38] Ji W-Y Liu D-L. Vaccination coverage survey of children aged 1–3 years in Beijing, China, 2005–2021. Vaccine. (2023) 41(43):6444–52. 10.1016/j.vaccine.2023.08.015 37709591

[39] Nigus M Zelalem M. Implementing nationwide measles supplemental immunization activities in Ethiopia in the context of COVID-19: process and lessons learnt. Pan Afr Med J. (2020) 37(Suppl 1):36. 10.11604/pamj.supp.2020.37.1.26614 33456660 PMC7796832

[40] Abatemam H Wordofa MA Worku BT. Missed opportunity for routine vaccination and associated factors among children aged 0–23 months in public health facilities of Jimma Town. PLOS Glob Public Health. (2023) 3(7):e0001819. 37490474 10.1371/journal.pgph.0001819 PMC10368238

[41] Michels SY Freeman RE. Evaluating vaccination coverage and timeliness in American Indian/Alaska native and non-Hispanic white children using state immunization information system data, 2015–2017. Prev Med Rep. (2022) 27:101817. 10.1016/j.pmedr.2022.101817 35656223 PMC9152883

[42] Koulidiati J-L Kaboré R. Timely completion of childhood vaccination and its predictors in Burkina Faso. Vaccine. (2022) 04(057):3356–65. 10.1016/j.vaccine.2022.04.057 35487810

[43] Fadiel Abu SA. Contributing factors of incomplete childhood primary vaccination, Sudan. Int J Med Res Health Sci. (2021) 10(3):53–61.

[44] Mekonnen AG Bayleyegn AD Ayele ET. Immunization coverage of 12–23 months old children and its associated factors in Minjar-Shenkora district, Ethiopia: a community-based study. BMC Pediatr. (2019) 19:1–8. 10.1186/s12887-019-1575-7 31200690 PMC6567439

[45] Girmay A Dadi AF. Full immunization coverage and associated factors among children aged 12–23 months in a hard-to-reach areas of Ethiopia. Int J Pediatr. (2019) 2019(1):1924941. 10.1155/2019/1924941 31263502 PMC6556785

[46] Golla EB. Vaccination dropout and associated factors among children in Ethiopia: a systematic review and meta-analysis (2014–2024). BMC Pediatr. (2025) 25(2025):426. 10.1186/s12887-025-05786-3 40426069 PMC12117780

[47] Yadita ZS Ayehubizu LM. Full immunization coverage and associated factors among children aged 12–23 months in Somali region, eastern Ethiopia. PLoS One. (2021) 16(12):e0260258. 10.1371/journal.pone.0260258 34874949 PMC8651113

[48] Adeloye D Jacobs W Amuta AO Ogundipe O Mosaku O Gadanya MA Coverage and determinants of childhood immunization in Nigeria: a systematic review and meta-analysis. Vaccine. (2017) 35(22):2871–81. 10.1016/j.vaccine.2017.04.034 28438406

[49] Woudneh AF Shiferaw NT. Exploring determinants of vaccination status among pediatric populations in East Gojam, Amhara Region, Ethiopia. BMC Pediatr. (2024) 24(1):763. 10.1186/s12887-024-05256-2 39580386 PMC11585093

[50] Shumba C Maina R Mbuthia G Kimani R Mbugua S Shah S Reorienting nurturing care for early childhood development during the COVID-19 pandemic in Kenya: a review. Int J Environ Res Public Health. (2020) 17(19):7028. 10.3390/ijerph17197028 32992966 PMC7579158

