# Supplementary material for: Determinants of vaccination coverage during the COVID-19 pandemic among children aged 12–23 months in southern Ethiopia: a cross-sectional study
Source: Front Pediatr. 2025 Aug 22;13:1566443. doi: 10.3389/fped.2025.1566443 (PMC12411534; doi:10.3389/fped.2025.1566443)
Supplement: Supplementary file 1 [file Datasheet1.pdf]

Determinants of vaccination coverage during Covid-19 pandemic among children aged 12-23 months in southern Ethiopia, 2021: a cross-sectional study

STROBE Statement—checklist of items that should be included in reports of observational studies

	Item no.	Recommendation	Status
Title and abstract	1	(a) Indicate the study's design with a commonly used term in the title or the abstract	The design is indicated in the title
		(b) Provide in the abstract an informative and balanced summary of what was done and what was found	Abstract paragraph 2 & 3
Introduction			
Background/rationale	2	Explain the scientific background and rationale for the investigation being reported	Page 3 to 5<br>Rationale: line 82 to 91
Objectives	3	State specific objectives, including any prespecified hypotheses	Line 91 & 92, Page 5
Methods			
Study design	4	Present key elements of study design early in the paper	Line 95 to 96,page 5
Setting	5	Describe the setting, locations, and relevant dates, including periods of recruitment, exposure, follow-up, and data collection	Setting a& location: Line 96 to 98<br>Data collection: page 7 to 8
Participants	6	(a) Give the eligibility criteria, and the sources and methods of selection of participants	Line 104 to 125 , page 6 to 7
Variables	7	Clearly define all outcomes, exposures, predictors, potential confounders, and effect modifiers. Give diagnostic criteria, if applicable	Line 127 to 134 ,page 7 and<br>line 151 to 158, page 8
Data sources/ measurement	8*	For each variable of interest, give sources of data and details of methods of assessment (Measurement). Describe comparability of assessment methods if there is more than one group	Line 136 to 143, page 7<br>line 151 to 158,page 8
Bias	9	Describe any efforts to address potential sources of bias	Line 143 page 7
Study size	10	Explain how the study size was arrived at	Line 114 to 118, page 6
Quantitative variables	11	Explain how quantitative variables were handled in the analyses. If applicable, describe which groupings were chosen and why	The appropriate statistical model used and in the bivariate model variables with p value of 25% were chosen in order not to miss epidemiologically important variables.

			Data processing & analysis, Line 160 to 168 ,page 8
Statistical methods	12	(a) Describe all statistical methods including those used to control for confounding	First bivariate logistic regression was done then using a p value of <25% the second model multivariate logistic regression done line 165 to 167, page 8
		(b) Describe any methods used to examine subgroups and interactions	Not done
		(c) Explain how missing data were addressed	It was not a concern as the response rate was enough to analyze the data
		(d) If applicable, describe analytical methods taking account of sampling strategy	Not applicable
		(e) Describe any sensitivity analyses	Not applicable
Results			
Participants	13*	(a) Report numbers of individuals at each stage of study—e.g. numbers potentially eligible, examined for eligibility, confirmed eligible, included in the study, completing follow-up and analyzed	Result section:<br>Socio-demographic characteristics line 172 to 175 page 10<br>Vaccination coverage line 177 to 182 page 10<br>Factors predicting vaccination coverage line 184 to 200 page 10 & 11
		(b) Give reasons for non-participation at each stage	Not applicable
		(c) Consider use of a flow diagram	Not applicable
Descriptive data	14*	(a) Give characteristics of study participants (e.g. demographic, clinical, social) and information on exposures and potential confounders	Result section:<br>Socio-demographic characteristics line 172 to 175 page 10<br>Vaccination coverage line 177 to 182 page 10
		(b) Indicate number of participants with missing data for each variable of interest	Not applicable
Outcome data	15*	Report numbers of outcome events or summary measures	Result section:<br>Vaccination coverage line 177 to 178 page 10
Main results	16	(a) Give unadjusted estimates and, if applicable, confounder-adjusted estimates and their precision (e.g., 95% confidence interval). Make clear which confounders were adjusted for and why they were included	Factors predicting vaccination coverage Line 184 to 200 page

		(b) Report category boundaries when continuous variables were categorized	Not applicable
		(c) If relevant, consider translating estimates of relative risk into absolute risk for a meaningful time period	Not applicable
Other analyses	17	Report other analyses done—e.g. analyses of subgroups and interactions, and sensitivity analyses	Not done
Discussion			
Key results	18	Summaries key results with reference to study objectives	Discussion section It is well stated in line 202 to 266 page 11 to 14<br>Conclusion and recommendation section: It is summarized in line 281 to 286 page 15
Limitations	19	Discuss limitations of the study, taking into account sources of potential bias or imprecision. Discuss both direction and magnitude of any potential bias	Well stated in line 273 to 278 page 14
Interpretation	20	Give a cautious overall interpretation of results considering objectives, limitations, multiplicity of analyses, results from similar studies and other relevant evidence	Discussion section It is well stated in line 202 to 277 page 11 to 14
Generalizability	21	Discuss the generalizability (external validity) of the study results	It is well addressed as specific population included, a national DHS tool adapted and vaccination coverage cross-checked with child's vaccination card
Other information			
Funding	22	Give the source of funding and the role of the funders for the present study and, if applicable, for the original study on which the present article is based	No funding was received to conduct this study. Line 330

*Give information separately for exposed and unexposed groups.

Note: An Explanation and Elaboration article discusses each checklist item and gives methodological background and published examples of transparent reporting. The STROBE checklist is best used in conjunction with this article (freely available on the Web sites of PLoS Medicine at http://www.plosmedicine.org/, Annals of Internal Medicine at http://www.annals.org/, and Epidemiology at http://www.epidem.com/). Information on the STROBE Initiative is available at www.strobe-statement.org.
