# Supplementary material for: Determinants of vaccination coverage during the COVID-19 pandemic among children aged 12–23 months in southern Ethiopia: a cross-sectional study
Source: Front Pediatr. 2025 Aug 22;13:1566443. doi: 10.3389/fped.2025.1566443 (PMC12411534; doi:10.3389/fped.2025.1566443)
Supplement: Supplementary file 2 [file Datasheet2.pdf]

Title: Title of the study: Determinants of vaccination coverage during Covid-19 pandemic among children aged 12-23 months in southern Ethiopia: a cross-sectional study<br>Form 1: Questionnaire for mothers of children age 12-23 months<br>*This form has to be filled only for children aged between 12-23 months*			
**Section 1: Identification and Consent**			
**101**	Questionnaire ID	________________________	
**102**	Area Identification	Zone___________________Town_______________	
**103**	Area of residence	Urban………………………………….1<br>Rural…………………………………..2	
**104**	Personnel	a) Interviewer___________Interviewer code__________<br>b) Field Supervisor________Supervisor code__________	
**105**	Date of visit	[_____|_____| ______| DD |MM |YYYY	
**T1**	Time at beginning of interview	____:____	

Introduction and Consent

Hello. My name is ____________________ and I am here as a part of data collector being conducted by Tigist Enyew and her co authors. The information obtained from you will be used to design EPI interventions in your locality as well as nationally. The interview will take approximately 30 minutes. All the information obtained from you will be kept confidential.

Your participation in the study is completely voluntary. You may withdraw your consent and discontinue participation at any time or you have the right not to answer any question that you do not want to. However, I hope you will participate in the survey since your views are important.

Do you want to ask me anything about the study?

May I begin the interview now? 1. [ ] Yes. 2. [ ] No

Signature of interviewer____________________ Date ____/____ /2021

Section 2: Background and Household Characteristics

Sr.No	Prompt	Response	Skip
**201**	What is the NAME of your child aged 12-23 months?	____	
**202**	Sex of Child (NAME)?	Boy………..1<br>Girl…..……..2	
**203**	What is the age of your child (NAME)?<br>**Record Age In Completed Months**	______________ Month	
**204**	What is the birth date of the child (NAME)?	[______/______/______]<br>Day / Month / Year	
**205**	Verify Child's Date Of Birth By Asking To See The Child's Health Card / Immunization Card Or Birth Certificate	Child's date of birth verified……………...1<br>Not possible to verify……………………..2	
**206**	What is the birth order of the child (NAME) (first, second, etc)?	First.........................1<br>Second...................2<br>Third........................3<br>Other specify_____________	**If 1 skip to 209**
**207**	What is the age of the child younger than (NAME)?<br>**Record Age In Completed Months**<br>**If No Younger Child Record "99"**	Number of months [___|___]	
**208**	What is the age of the child elder than (NAME)?<br>**Record Age In Completed Months**<br>**If No Elder Child Record "99"**	Number of months [___|___]	
**209**	Who is the primary care taker of the child (NAME)?	Mother………………………………..1<br>Father…………………………………2<br>Both …………………………….…3<br>Other than parents……….…………4	
**210**	Is primary care taker responding?	Yes……………………..…….1	

	**Don't Ask, Just Record**	No……………………..….2	
211	What is the age of the primary care taker?	Age in years………..<br>Don't Know age………….99	
212	Is the primary care taker able to read or write?	Yes……………………..…….1<br>No……………………..….2	 **→215**
213	Did the primary care taker ever attend formal school?	Yes………………………….1<br>No………………………..…….2	 **→215**
214	What is the highest grade the primary care taker completed?	Elementary school…………...………1<br>High school......………….…………..2<br>Technical/vocational certificate . . . .3<br>University/college diploma …………4<br>University/college degree or Higher . . . 5	
215	What is the religion of the primary care taker?	Orthodox …………………………….1<br>Catholic . . . . 2<br>Protestant . . . . 3<br>Muslim . . . . 4<br>Other(Specify)_________________	
216	Marital status	Married…………… 1<br>Unmarried . . . . 2<br>Divorced……………………….3<br>separated . . . . 4<br>widowed……………………5<br>Living together…………………….6	 **→218**
217	Is your husband/partner living with you now or is he staying elsewhere?	Living together . . . . 1<br>Staying elsewhere. 2	
218	How many times you have pregnant? (including those that did not end with a live births)	Number _________	
219	How many times you have given live birth?	Boys _____ Girls ____Total_______	
220	How long does it take you to walk to the nearest health post?<br>**If Less Than One Hour, Record In Minutes**<br>***No Health Post In The Keble; Record "00"***	Minutes _________Hours _________	
221	How long does it take you to walk to the nearest health center?<br>**If Less Than One Hour, Record In Minutes**<br>***No Health center In The Keble; Record "00"***	Minutes _________Hours _________	
222	How long does it take you to walk to the immunization site?<br>**If Less Than One Hour, Record In Minutes**	Minutes _________Hours _________<br>Don't know the time required to reach…..88<br>Don't know the immunization site……………99	

Section 3: Child Immunization

301	Do you have a card/paper where (NAME's) vaccinations are written down?<br>**If Yes, May I See It?**	Yes................................ 1<br>No ....2<br>Don't Know……….99	**→303**
302	Did you ever have a vaccination card/paper for (NAME)?	Yes................................ 1<br>No ....2<br>Don't Know……….99	 **→306**
303	Does the child have a scar from BCG vaccination?<br>**Check For BCG Scar**	Yes................................ 1<br>No 2	
304	Copy vaccination date for each vaccine from the card/paper<br><br>**WRITE "44" IN "DAY" COLUMN IF CARD SHOWS THAT A VACCINATION WAS GIVEN, BUT NO DATE IS RECORDED**<br><br>a) BCG<br>b) Polio 0<br>c) Polio 1<br>d) Polio 2	Day Month Year<br><br>BCG [___|___][___|___][___||___]<br>Polio 0 [___|___][___|___][___||___]<br>Polio 1 [___|___][___|___][___||___]	 <br><br><br><br><br><br>**If fully vaccinated,**

	e) Polio 3<br>f) Penta 1<br>g) Penta2<br>h) Penta3<br>i) PCV1<br>j) PCV2<br>k) PCV3<br>l) Rota1<br>m) Rota2<br>n) Measles	Polio 2 [___|___][___|___][___||___]<br>Polio 3 [___|___][___|___][___||___]<br>Penta1 [___|___][___|___][___||___]<br>Penta2 [___|___][___|___][___||___]<br>Penta3 [___|___][___|___][___||___]<br>PCV1 [___|___][___|___][___||___]<br>PCV2 [___|___][___|___][___||___]<br>PCV3 [___|___][___|___][___||___]<br>Rota1 [___|___][___|___][___||___]<br>Rota2 [___|___][___|___][___||___]<br>Measles [___|___][___|___][___||___]	**then go to 310**
**305**	Has (NAME) received any vaccinations that are not recorded on this card/paper, including vaccinations received in a national immunization day campaign?	Yes…………….1<br>No..…………..2	
**306**	Did (NAME) ever receive any vaccinations to prevent him/her from getting diseases, including vaccinations received in a national immunization day campaign?	Yes................................ 1<br>No ....2<br>Don't know......................... 99	**→309**<br>**→309**
**307**	Is the child (NAME) vaccinated during campaign or routine?	Routine…..……………….1<br>Campaign……………………2<br>Don't know……………………99	
**308**	Please tell me if (NAME) received any of the following vaccinations:	**Read questions 308a – 308g**	
**308a**	A BCG vaccination against tuberculosis that is, an injection in the arm or shoulder that usually causes a scar?	Yes................................ 1<br>No ....2<br>Don't know......................... 99	
**308 X**	**CHECK FOR BCG SCAR.**	Yes................................ 1<br>No 2	
**308b**	Polio vaccine, that is, drops in the mouth?	Yes................................ 1<br>No ....2<br>Don't know......................... 99	**→308e**<br>**→308e**
**308c**	When was the first polio vaccine received, just after birth or later?	Just after birth…………….1<br>Later..…………..2	
**308d**	How many times was the polio vaccine received?	Number……..[___|___]<br>Don't Know……………...99	
**308e**	PENTA/DPT vaccination, that is, an injection given in the left thigh sometimes at the same time as polio drops	Yes................................ 1<br>No ....2<br>Don't know......................... 99	**→308g**<br>**→308g**
**308f**	How many times was the PENTA/DPT vaccine received?	Number……..[___|___]<br>Don't Know…..99	
**308g**	PCV vaccination, that is, an injection given in the right thigh sometimes at the same time as polio drops and PENTA injection.	Yes................................ 1<br>No ....2<br>Don't know......................... 99	**→308i**<br>**→308i**
**308h**	How many times was the PCV vaccine received?	Number……..[___|___]<br>Don't Know…..99	
**308i**	Rota vaccine, that is, drops in the mouth to prevent diarrhea?	Yes................................ 1<br>No ....2<br>Don't know......................... 99	**→308k**<br>**→308k**
**308j**	How many times was the Rota vaccine received?	Number……..[___|___]<br>Don't Know……………..99	
**308k**	An injection on the left upper arm to prevent measles, given around 9 months of age?	Yes................................ 1<br>No ....2<br>Don't know......................... 99	
**309**	Look back at the information on the child's immunization card or the information given by the mother<br><br>**If Child Never Immunized Or Not Fully Immunized, Ask The Following <u>Question</u>:**<br>Why the child was not fully immunized?<br>**Without Probing, Circle All Responses Mentioned**<br>**Mentioned (M)=1**	**<u>M=1 NM=2</u>**<br>**Lack of Information**<br>Unaware of need for immunization…1 2<br>Unaware of need to return for subsequent dose…1 2<br>Place and/or time of immunization unknown……1 2<br>Fear of side effects………1 2<br>Wrong ideas about contra-indications…1 2<br>**Lack of motivation**<br>Postponed until another time……1 2	

	**Not Mentioned (NM)=2**	No faith in immunization……………1 2<br>Rumors……………………………1 2<br>**Obstacles**<br>Place of immunization too far………..1 2<br>Time of immunization inconvenient…1 2<br>Vaccinators absent……………………..1 2<br>Vaccine not available………………...1 2<br>Mother too busy………………………1 2<br>Family problem, including illness of the mother…………………………………1 2<br>Child ill-- not brought………………1 2<br>Child ill—brought but not given immunization.... 1 2<br>Long Waiting time…………………..1 2<br>Other Specify ……..……………………………<br>**Covid-19 Pandemic**<br>full lockdown of immunization services 1 2<br>Physical distancing measures 1 2<br>stay at home messaging 1 2<br>fear of mothers & caregivers to visit health facilities1 2<br>closing of borders for vaccination service access 1 2<br>diversion of resources for the pandemic 1 2<br>postponed vaccination campaigns1 2<br>Other, specify _______________	
**310**	If the child received any routine vaccination, where did (NAME) receive last routine vaccination?	Hospital………………………..1<br>Health center…………………2<br>Health post……………………3<br>Private clinic……………………..4<br>Outreach……………………………..5<br>Other specify……………………………6<br>Don't know………………………………99	
**311**	When you were pregnant (NAME) did you go to a health facility for antenatal care?	Yes .... 1<br>No .... 2<br>Don't remember……………99	 **→313**
**312**	How many times did you receive antenatal care in the health facility during (NAME) pregnancy?	Number [_____|_____]<br>Don't Know…………………..99	
**313**	Have you ever been to a health facility for any purpose other than for vaccination (e.g. child was sick, sibling visit)	Yes .... 1<br>No .... 2<br>Don't remember……………99	 **→315**<br>**→315**
**314**	Have your child ever received vaccines when you went to health facility for a reason other than child vaccination?	Yes .... 1<br>No .... 2<br>Don't remember……………99	
**315**	Have you ever refused vaccination for this child?	Yes .... 1<br>No .... 2<br>Don't remember……………99	 **→401**<br>**→401**
**316**	Why did you ever refuse for vaccination?<br><br>**Without Probing, Circle All Responses Mentioned**	**Yes No**<br>Too many shoots at visit ………1 2<br>Child ill……………………1 2<br>Wait too long, so left………………….. 1 2<br>Did not like health worker……………….. 1 2<br>Other, specify ____________________<br>Do not know ………………………………99	
	**Section 4: Knowledge and Awareness**		
**401**	Were you told about side effects or problems your child might have with vaccines?	Yes……………1<br>No..…………...2	
**402**	Were you told what to do if your child experienced side effects?	Yes……………1<br>No..…………...2	
**403**	In the last three months have you heard about vaccinations by any of the following means?<br><br>**Read Out The List**<br>**Circle "1" For Yes, And "2" For No.**	**Yes No**<br>Radio……………………………….. 1 2<br>Television.…………………………. 1 2<br>Newspaper/magazine……………….. 1 2<br>Pamphlet/poster…....……………….. 1 2<br>Health worker……………..……….. 1 2<br>Community events………..……….. 1 2<br>Health development armies....…….. 1 2	

		Health Extension Worker…..…..…...1 2<br>Other , Specify ____________________	
**404**	What messages have you heard about immunization?<br>**Probe And Mark All That Apply**	About campaigns………………1<br>Importance of vaccination………………2<br>Where to get routine vaccination……………3<br>Age to get routine vaccination………………4<br>Return to next doses of the routine vaccination……………………………………………5<br>About new vaccinations (pneumococcal/ Rota vaccine)…………………………………..……..…6<br>Other, specify________________________<br>Don't know…………………………99	
**405**	When do you think a child should start immunization?	[_____|_____] Month	
**406**	Do you think immunization prevents against childhood illnesses?	Yes................................ 1<br>No ....2<br>Don't know......................... 99	
**407**	Did you seek advice or treatment when your child gets sick from any source (health facility, holly water, traditional healer etc)?	Yes................................ 1<br>No 2	
**408**	When your child is sick, where would/do you take the child?<br><br>**Without Probing, Circle All Responses That Apply**	Health facility……………………1<br>Holly water…………………..2<br>Traditional healer…………………3<br>Prayer place……………………4<br>Other, specify ______________<br>Don't know………………………………99	
**409**	When your child is sick, where would/do you take the child first?	Health facility……………………1<br>Holly water…………………..2<br>Traditional healer…………………3<br>Prayer place……………………4<br>Other, specify ______________<br>Don't know………………………………99	

THANK YOU!!
